# Hyperbaric Oxygen Treatment of Keratitis Following Facial Hyaluronic Acid Injection

**DOI:** 10.31486/toj.18.0133

**Published:** 2020

**Authors:** Nancy Worley, Mary Lupo, Kate Holcomb, Ginny Kullman, Ebrahim Elahi, Jasmine Elison

**Affiliations:** ^1^ Tulane University School of Medicine, New Orleans, LA; ^2^ Lupo Center for Aesthetic and General Dermatology, New Orleans, LA; ^3^ Pure Dermatology, Metairie, LA; ^4^ Department of Ophthalmology, Ochsner Clinic Foundation, New Orleans, LA; ^5^ Department of Ophthalmology, Mount Sinai School of Medicine, New York, NY; ^6^ Retina and Vitreous Specialists New Orleans, Metairie, LA

**Keywords:** *Dermal fillers*, *hyaluronic acid*, *hyperbaric oxygenation*, *keratitis*

## Abstract

**Background:** With the increasing popularity of facial filler injections, growing numbers of complications have been reported.

**Case Report:** We present the case of a 60-year-old female with vision changes and keratitis following hyaluronic acid (HA) facial filler injections who completely recovered following hyperbaric oxygen treatment (HBOT).

**Conclusion:** Using HBOT to successfully treat ocular ischemia has been reported, but to our knowledge, our case is the first report of successful HBOT use for ocular ischemia and keratitis following cosmetic facial HA injection.

## INTRODUCTION

Cosmetic facial filler injections are an increasingly popular method of facial rejuvenation. Of the 2.4 million soft tissue filler procedures performed in the United States in 2015, hyaluronic acid (HA) injections accounted for more than 1.9 million of them, an 8% increase from 2014.^1^ HA facial fillers are sterile, biodegradable, biocompatible, and reversible with hyaluronidase. Although HA injection is generally perceived as a safe minor procedure, a range of adverse events may occur. Reported complications associated with dermal fillers include bruising, edema, skin discoloration, infection, nodular masses, paresthesia, vascular compromise, vision changes, cerebral infarction, and even blindness.^2^ We present the case of an unusual keratitis following facial HA injection that was successfully managed with the use of hyperbaric oxygen treatment (HBOT).

## CASE REPORT

A 60-year-old Caucasian female with no significant medical history received 2 HA injections (a Restylane Silk injection and a Restylane Lyft injection, Galderma Laboratories, LP) in the horizontal nasal crease, maxilla, and nasolabial folds. She had received HA injections in the same facial areas without complication 9 months earlier. Two days after the injections, she was treated for ecchymosis with a 532 nm laser. The next day, she experienced pain in the left nasolabial fold and left eye and reported blurred vision in the left eye. She presented to the emergency department; an optometrist diagnosed her with keratitis and started her on erythromycin ointment twice daily in the affected eye.

**Figure 1.**
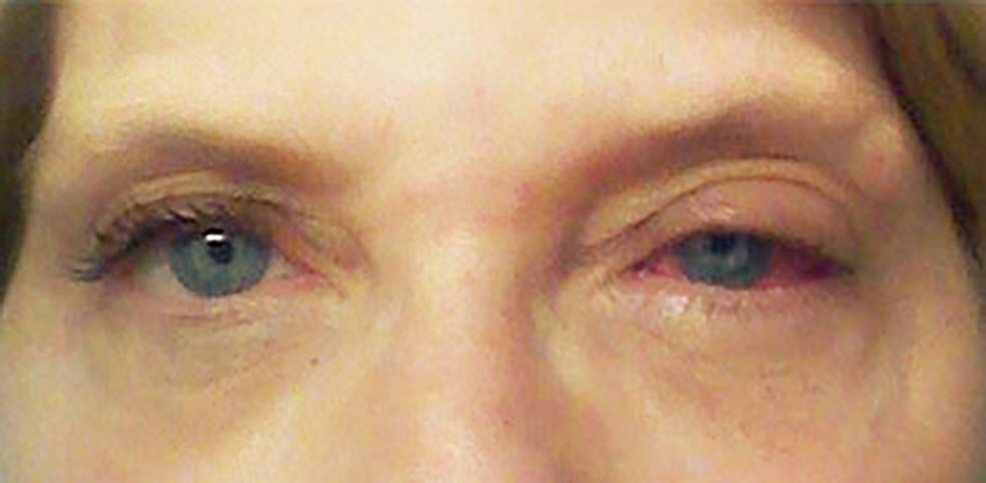
**The patient had reticulate patches in the skin over the left glabellar region, as well as left upper eyelid edema and conjunctival injection.**

The following day, the patient returned to the dermatologist's office and reported increasing pain in the left cheek and worsening redness, irritation, and continued vision loss in the left eye. She described severe pressure and pain on the left nasal crease extending up the left side of the nose and to the globe of the eye. She had a 1 cm reticulate patch and a firm nonvisible 3.5 × 1.5 cm area of swelling on palpation in the glabellar region (Figure 1). She was treated with 450 units of hyaluronidase (Hylenex, Halozyme Therapeutics, Inc.) to the nasolabial fold, the left side of the nose, and the glabella. She was also given aspirin 81 mg and ibuprofen 800 mg. She reported an 80% improvement in the pressure and pain within 15 minutes after administration of the hyaluronidase. She was instructed to apply 2% nitroglycerine ointment to the affected areas and was referred to an ophthalmologist for the evaluation of her decline in vision.

**Figure 2.**
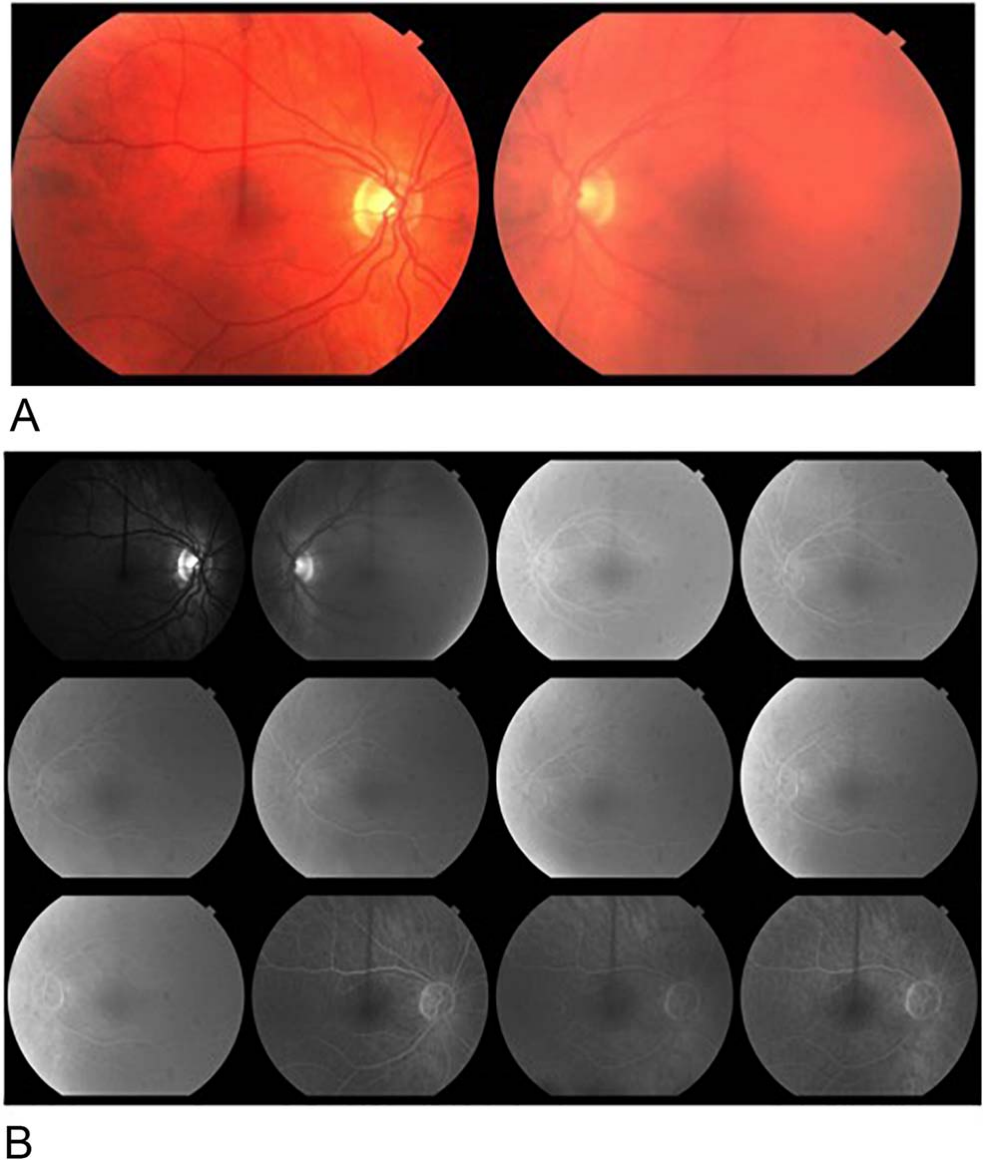
**(A) Retinal examination was normal with media opacity in the left eye because of keratitis. (B) Fluorescein angiography demonstrated full vascular filling in both retinas.**

The patient had an ocular history of dry eye, for which she had been using cyclosporine until the day of the injections. She had no history of contact lens use, herpetic eye disease, or recent rashes. Ocular medication was erythromycin ointment that she had started one day prior. Snellen visual acuity with correction was 20/25 in the right eye and 20/125 in the left eye and 20/40 with pinhole. Right eye examination and pressures were within normal limits. Examination of the left eye revealed no afferent pupillary defect, full extraocular movements and confrontational fields, 1+ left upper lid edema, 2+ conjunctival injection, intraocular pressure of 20 mmHg, and stromal opacity (keratitis) nasally with punctate fluorescein staining. The anterior chamber showed no cells and trace flare, but regularly shaped globules suspended in the aqueous were present. Retinal examination was within normal limits, and fluorescein angiography showed full vascular filling with a hazy view because of media opacity (Figures 2A and 2B). The patient was advised to start ofloxacin antibiotic drops 4 times daily, continue erythromycin, and start cyclopentolate for discomfort.

**Figure 3.**
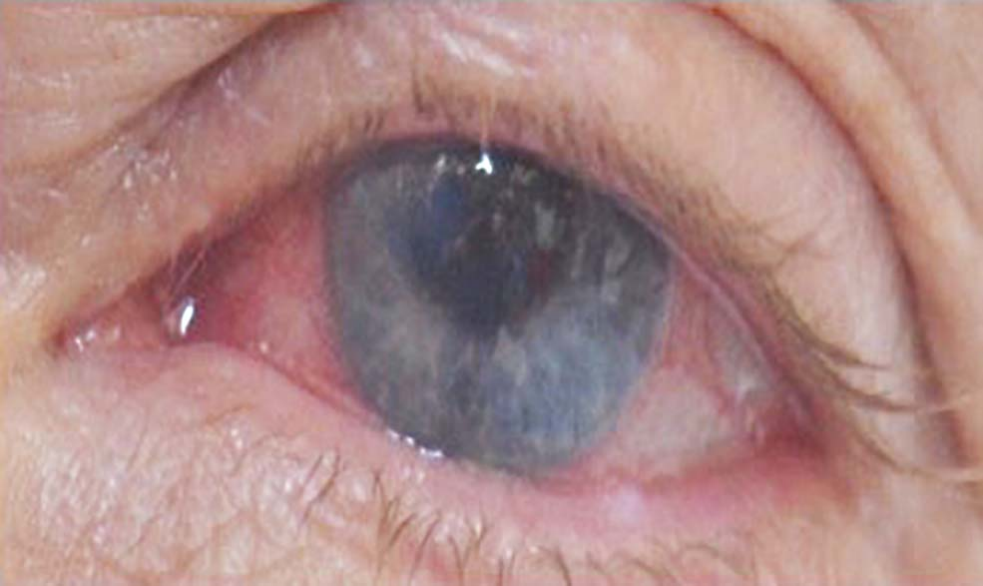
**Photograph taken before the first hyperbaric oxygen treatment shows conjunctival injection, nasal keratitis, and a lack of corneal clarity because of corneal edema of the left eye.**

Twelve hours later, the patient reported a dramatic decrease in her vision. Visual acuity with correction in the left eye was 20/200 with no improvement on pinhole testing. She had generalized 2+ corneal edema, sparing a small area of the temporal cornea. The anterior chamber clear globules were no longer visible, and the keratitis appeared unchanged with persistent punctate staining of the epithelium. The view of the fundus was hazier, but the retina and the retinal vasculature appeared normal, and intraocular pressure was in the normal range. The patient was treated with 75 units of hyaluronidase to the inferior periocular region and 75 units to the glabella and left nasal sidewall (150 units total). Because of her acute vision deterioration, she was referred for emergent HBOT for possible anterior segment ischemia and keratitis (Figure 3).

The patient underwent nine 90-minute HBOTs and stated that her vision began to improve within 20 minutes of the first session. Her visual acuity measured by near card in the hyperbaric facility improved from 20/200 at the start of treatment to 20/60 at the 20th minute. On day 2 of her HBOT, the patient's visual acuity was 20/60, and intraocular pressure was 12 mmHg. Corneal edema and keratitis were significantly decreased; the area of stippled staining was smaller, approximately 2 × 1.5 mm; the anterior chamber was clear; and the fundoscopic examination was normal.

**Figure 4.**
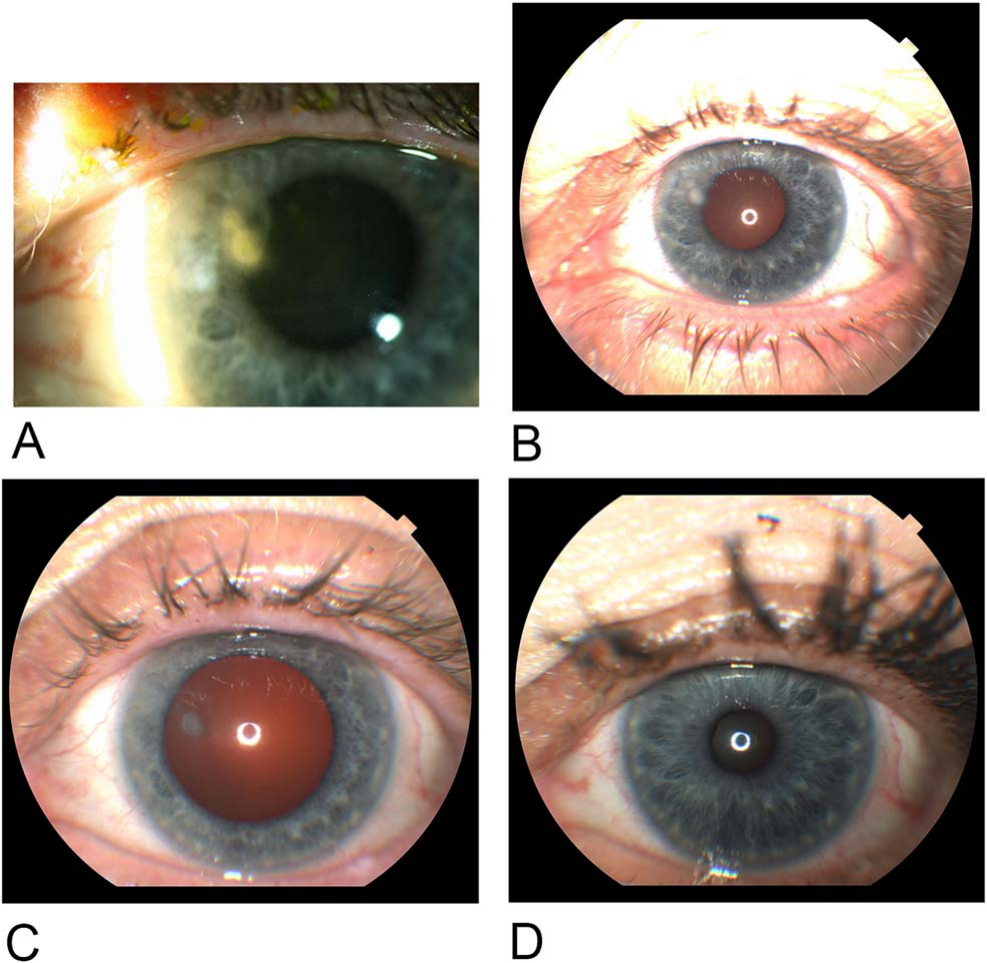
**(A and B) Small corneal ulcer and adjacent keratitis on day 3. (C) Stromal scar and resolving corneal ulcer on day 10. (D) Complete resolution 1 month after hyperbaric oxygen treatment.**

On day 3, the patient stated that her vision fluctuated but was gradually improving overall. She perceived that her vision improved during and immediately after each HBOT but that the effects of each treatment had decreased by the time she had the next session. Visual acuity was 20/200 in the left eye and 20/60 with pinhole. The keratitis was nearly resolved, but a corneal infiltrate measured 1.5 × 1.5 mm (Figures 4A and 4B). The patient was started on fortified vancomycin 25 mg/mL and tobramycin 15 mg/mL drops 4 times daily, GenTeal ointment (Novartis Pharmaceuticals Corp.) every night, and valacyclovir (Valtrex) 500 mg 3 times daily to account for the possibility of an atypical herpetic component.

She continued to improve with each 90-minute HBOT. On day 10, her vision was 20/20, and the infiltrate had nearly resolved (Figure 4C). A small stromal scar, approximately 1 mm in diameter, corresponded to the site of the ulcer in the nasal cornea. One month later, the patient reported that her vision had returned to baseline. Her visual acuity with correction was 20/20. The cornea was clear, and her eye examination was normal (Figure 4D).

## DISCUSSION

While reports of retinal vascular occlusions following facial HA injections have been increasingly numerous,^3-7^ to our knowledge, ours is the first case of anterior segment and corneal pathology following facial HA injection that resolved with HBOT. The unusual and aggressive keratitis that occurred in our patient after facial HA injection responded quickly to HBOT, suggesting a reversible ischemic process. The keratitis occurred on the same side as the facial pain ecchymosis and skin reticulation post HA injection, indicating a likely association between the ocular findings and the injection.

The phenomenon of HA emboli to the eye is possible because branches of the ophthalmic artery are part of an extensive anastomotic network that communicates with the facial vasculature, including the supraorbital, supratrochlear, and dorsal nasal arteries.^8^ The pathways and connections between these vessels may vary between patients, and slight differences in anatomy may make certain vessels more prone to emboli than others. We believe that the underlying pathologic cause in our patient may have been a tiny embolus of HA lodged in a portion of the angular artery to dorsal nasal artery; the impact could have been gradual if the vessel was only partially plugged, leading to the delayed onset of skin findings and pain.

The cause of the corneal findings is less clear because the cornea is an avascular structure and thus theoretically fairly resistant to localized ischemic events. We hypothesize the presence of emboli causing low-grade ocular ischemia that could have led to the keratitis and corneal edema. When the hyaluronidase was injected, the plug was dissolved and may have diffused into the anterior chamber, possibly causing a toxic anterior segment syndrome that may have contributed to the acute and severe corneal edema the patient experienced the following morning.

The immediate improvement and gradual complete recovery of the eye in response to HBOT are suggestive of ischemia. This patient had rapid improvement with each treatment that was partially sustained until her next session. While we would expect an ischemic injury to the cornea to take many hours or days to heal, even with HBOT, our patient had marked improvement in minutes. Sustained improvement, however, took more than a week.

This stepwise but gradual improvement is consistent with corneal physiology. The cornea is constantly absorbing aqueous humor but maintains its clarity via the endothelial pump mechanism. The functioning of this mitochondrial-rich endothelial cell layer is highly dependent on oxygen. A hypoxic environment results in corneal edema and loss of clarity. Reversal of the hypoxic environment and hyperoxygenation of the cornea reactivates the pump mechanism and decreases edema. Oxygenation of the aqueous humor can occur through direct diffusion across the cornea or through the arterioles in the limbal circulation, palpebral conjunctiva, or blood supply to the ciliary body, all of which would be greatly improved during HBOT.^9^ The implication is that the reduction of corneal edema likely led to the patient's initial rapid improvement, and a slower amelioration of her underlying ischemic process occurred with each treatment. Without the use of steroids, the keratitis gradually resolved over the course of 9 HBOT sessions, as did the small corneal ulcer that had developed.

While the exact mechanism of visual recovery in this case can only be postulated, the sequence of events clearly suggests that an ischemic process may have directly or indirectly led to the corneal findings. The presence of anterior chamber particulate matter is puzzling and points to a possible retrograde flow of material through the arteriole circulation. Because no tissue was obtained in this case, this theory remains unconfirmed.

The glabellar area may be particularly high risk because of its vascular anatomy and proximity to the retinal arterial circulation. Minimizing injection volume and the use of cannulas vs needles may decrease the inadvertent retrograde injection of dermal fillers in this facial region.

## CONCLUSION

With the increasing popularity of facial filler injections, the numbers of reported complications are growing. Our patient had vision changes and keratitis secondary to HA facial filler injections and completely recovered following HBOT. To our knowledge, this report is the first case of successful HBOT for ocular ischemia and keratitis following cosmetic facial HA injection.

## Notes

This article meets the Accreditation Council for Graduate Medical Education and the American Board of Medical Specialties Maintenance of Certification competencies for Patient Care and Medical Knowledge.
